# Characterization of kinesthetic motor imagery compared with visual motor imageries

**DOI:** 10.1038/s41598-021-82241-0

**Published:** 2021-02-12

**Authors:** Yu Jin Yang, Eun Jeong Jeon, June Sic Kim, Chun Kee Chung

**Affiliations:** 1 Department of Brain and Cognitive Sciences, Seoul National University College of Natural Sciences, Seoul, 08826 Republic of Korea; 2 The Research Institute of Basic Sciences, Seoul National University, Seoul, Republic of Korea; 3 Department of Neurosurgery, Seoul National University Hospital, Seoul, Republic of Korea

**Keywords:** Computational neuroscience, Sensorimotor processing

## Abstract

Motor imagery (MI) is the only way for disabled subjects to robustly use a robot arm with a brain-machine interface. There are two main types of MI. Kinesthetic motor imagery (KMI) is proprioceptive (OR somato-) sensory imagination and Visual motor imagery (VMI) represents a visualization of the corresponding movement incorporating the visual network. Because these imagery tactics may use different networks, we hypothesized that the connectivity measures could characterize the two imageries better than the local activity. Electroencephalography data were recorded. Subjects performed different conditions, including motor execution (ME), KMI, VMI, and visual observation (VO). We tried to classify the KMI and VMI by conventional power analysis and by the connectivity measures. The mean accuracies of the classification of the KMI and VMI were 98.5% and 99.29% by connectivity measures (alpha and beta, respectively), which were higher than those by the normalized power (p < 0.01, Wilcoxon paired rank test). Additionally, the connectivity patterns were correlated between the ME-KMI and between the VO-VMI. The degree centrality (DC) was significantly higher in the left-S1 at the alpha-band in the KMI than in the VMI. The MI could be well classified because the KMI recruits a similar network to the ME. These findings could contribute to MI training methods.

## Introduction

A brain machine interface (BMI) is designed to translate raw neural signals into motor commands so that it reproduces body movements with a neuroprosthetic device^1^. For people with a severe neuromuscular disability, a BMI might replace or even restore lost natural outputs^2^. In particular, one of the major goals for a BMI is to decode a command signal from the motor cortex, which serves as a new functional output^3^. Then the question is what is the command signal that disabled people can manipulate. The answer would be the neural activity while imagining a movement. Neural activity occurs even while a person imagines moving body parts. We call it motor imagery (MI), which is a particular type of mental imagery defined as the mental simulation of a specific action without any corresponding motor output^4^. The neural activity by MI is known to originate from the motor and somatosensory areas^5^. Thus, most of the movement BMIs use sensorimotor activities during MI. However, the network underlying a MI, or some kind of imagery can be different depending on the imagery tactics (e.g., object-related imagery activates object areas, whereas the imagery of faces activates face-related areas in the fusiform gyrus)^6^.

In the EEG-based BMI, about 15–30% of users do not gain control of the BMI system. This phenomenon in these subjects is called "BMI illiteracy"^7^, which may result from the individual variability of the EEG signals. The EEG environment may not be identical for each recording. Contact impedance, placement of the electrodes, and environmental noise slightly vary from time to time. Additionally, the EEG activity may also change according to the alertness, attention, and inherent fluctuation of a subject. Furthermore, this variability could be larger in an imagery task rather than in an actual movement task because the individual strategy of imagination may differ among the subjects. In addition, BMI illiteracy may not only be due to the user but also partly to the training protocol and the feedback^8^. Mastering a MI resembles the acquisition of a new motor skill^9^. As appropriate practice improves a motor skill, imagery practice by appropriate feedback is required for robust BMI control^8,10^.

Recently, several approaches have been tried such as somatosensory^11^, tactile^12^, and visual feedbacks^13^ to give a more natural feedback to the subject. These feedback approaches relied on accurate classification. However, there is a lack of accurate classification for the type of MI that the subject used.

There are two main types of motor imageries: kinesthetic motor imagery (KMI)^14^ and visual motor imagery (VMI)^14^. KMI is described as the ability to imagine performing a movement by means of having an impression of the muscle contraction and sensation during an actual movement. On the other hand, VMI is the ability to imagining seeing myself performing the movement. Because most of the decoders for motor prediction are designed based on an actual movement, KMI could be more effective than VMI. Thus, the use of a KMI would be better to improve the BMI performance. However, without specific instructions, subjects may be confused in the imagery tactics. Furthermore, people with motor disabilities had more difficulties with kinesthetic imagination compared to healthy participants^15^. It would be helpful if we could classify the imagery types and give feedback to the subject.

Previous EEG studies that recognized different mental states such as motor execution (ME), KMI, VMI, and visual observation (VO) showed that mental states could be classified with accuracies in KMI (67%), VMI (56%), ME (80%) and VO (80%)^14^. The classification features were extracted based on the strength of the spatial cortical activity (e.g., frequency bands and electrode sites). However, several studies reported that the brain activation patterns of KMI and VMI showed similar activation patterns with only a temporal difference. KMI showed motor-related activation first, while VMI showed visual-occipital activations at the early stage^16^. In a practical condition, it is hard to detect the temporal changes. Furthermore, the local activity alone may have a high probability of a false positive because motor areas also show spontaneous activity even not in imagery or movement tasks (e.g., at rest)^17^. Therefore, a more robust approach rather than an activation-based method is needed. Motor-related brain regions apart from the motor cortex are involved in MI tasks^18,19^. The connectivity within motor-related brain regions would be an alternative feature to classify MI strategies. A previous study showed that connectivity in the dorsolateral prefrontal cortex increased during movement planning^20^. The connectivity refers to the statistical dependency of signals from spatially separated brain areas during a task performance^21^. The connectivity between brain areas can provide more direct evidence about information exchange even if the activity of a brain region does not increase compared to the baseline^22^. In addition, the connectivity could possibly reflect the underlying structural organization of anatomical connections^23^ by utilizing patterns of connectivity among brain regions that represent functional brain networks. Accordingly, many studies on memory^22^, resting-state^17,24^, and cognitive impairments^25^ used connectivity measures to analyze cognitive activities.

Despite the importance of MI in BMI, there is a lack of accurate methods for classifying MI types and providing feedback. In order to address this gap, the present study classified two types of MI with a connectivity measure. The study hypothesized that KMI would show more similar connectivity patterns with ME than that of VMI. Moreover, the global and local characteristics of these network patterns will be different between KMI and VMI.

## Methods

### Participants

Eleven healthy subjects (7 females; aged 25.5 years old; STD = 2.71 years) were recruited for this study. All subjects were novices in BMI. We used the Edinburgh Handedness Inventory to collect data on the handedness of the subjects. All the subjects were right-handed, and the score on the Edinburgh Handedness Inventory was above 80 for all subjects (87.73 ± 6.17)^26^. The intrinsic abilities of two motor imagery types in all subjects were tested using the Kinesthetic and Visual Imagery Questionnaire (KVIQ-10)^27^. No subjects were excluded from the analysis because the subjects' KVIQ-10 scores were higher than the exclusion criteria of 2 (KMI = 3.24 ± 0.60; VMI = 3.42 ± 0.70). Prior to the experiment, the experimenter explained KMI and VMI and instructed subjects to use a specific imagery strategy according to the instructions. All subjects provided written informed consent to participate in the experiment. This study was performed in accordance with the Declaration of Helsinki and was approved by the Institutional Review Board (IRB) of Seoul National University Hospital (1605-136-765).

### Instructions and tasks

Before beginning the experiment, the experimenter explained the composition of the tasks and what the subject should do during the recording. The subjects, wearing an EEG cap and a 3D display device (VR BOSS XTREK, SMARTPIA, Yongin, South Korea), sat comfortably in a fixed chair without an armrest. The video shown on the 3D display device displayed the target or trajectory of the arm according to each condition. At the beginning of a task, subjects put their hands on both thighs. Subjects were asked to perform all the tasks using their dominant hand. The experimental paradigm consisted of the ME, KMI, VMI, and VO conditions. These conditions represent the typical strategies utilized for BMI. In the ME condition, subjects were asked to follow the movement of the robot arm as similar as possible, as shown in the virtual reality device. In the KMI task, the subjects were instructed to imagine the same movements with their own arm that coincided with the movement sequence in the ME task. While using KMI, the subjects were instructed to imagine the sensation as much as possible, which was a feeling that the subjects just before felt during the ME tasks, without muscle movement. In the VMI task, subjects were asked to imagine the arm movement visually and to exclude the kinesthetic sensation. Lastly, in the VO task, subjects were instructed just to keep watching the same video as the ME task.

### Experimental paradigm

**Figure 1:**
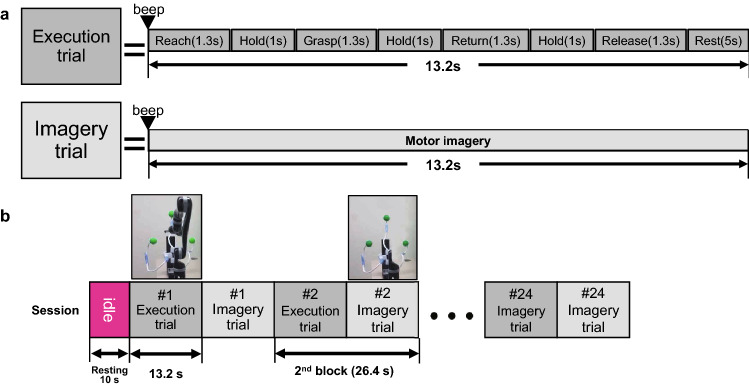
The graphic illustration of task paradigms. (**a**) The overall task paradigms of single trials as execution (ME, VO) and imagery (KMI, VMI) conditions. (**b**) The session design, which showed a moving robot arm when execution conditions and only target without a robot arm when imagery conditions.

For the sequence of tasks, movement imagery is difficult without any preceding practical task^28^. We designed the KMI tasks after the ME^14^. The KMI following the ME shows similar cortical activities^4,29^, which could be explained as a model of short-term memory that copies representational steps^4^. Likewise, the subjects performed the VMI tasks after VO. An experiment consisted of two tasks, which were pairs of ME-KMI and VO-VMI tasks. We showed a pre-recorded video manipulating a robot arm in every task. In the video for the ME and VO tasks, the robot arm sequentially performed reaching–hold–grasping–hold–return–hold–release for about 8.2 s and rest for about 5 s. In the imagery tasks for the KMI and VMI, the video displayed only a static target picture without a robot arm. Examples of the task design are briefly illustrated in Fig. 1. The reason why we removed the robot arm in the video for the imagery tasks was that the robot arm movement could disturb the MI with their own arm^30^. The targets in the video were placed in three directions, and the order of the targets was randomly assigned to avoid repeating the same direction as the previous block. A block consisted of an execution trial and an imagery trial, each of which took 13.2 s, taking a total of about 26.4 s. One session was composed of identical 24 blocks. The session consisting of 48 trials took about 11 min. We ran a total of four sessions that were two ME-KMI block sessions and two VO-VMI block sessions.

### Electrophysiological recordings and preprocessing

We acquired neural activities using a 64-channel EEG cap (Quich-CapEEG, Compumedics, Abbotsford, Victoria, Australia) and an amplifier (Neuroscan synamps2, Compumedics, Abbotsford, Victoria, Australia) with an additional electrode for a reference. The reference electrode was attached to the mastoid opposite the dominant hand of the subject. Movement of the arm was tracked by a motion capture system with an active optical marker (3D investigator, Northern Digital Inc., Waterloo, Ontario, Canada) during the neural signal recording. The sampling frequency of the signals was 2000 Hz for the EEG signal and 1000 Hz for the motion tracker. Both were resampled to the same frequency of 500 Hz. In order to synchronize the EEG amplifier and motion tracker, we delivered a 5 V input simultaneously to all devices before starting the sessions (e.g., through a transistor-transistor logic (TTL) port and the external trigger port of the 3D investigator amplifier).

### Source analysis and regions of interest (ROIs)

**Figure 2:**
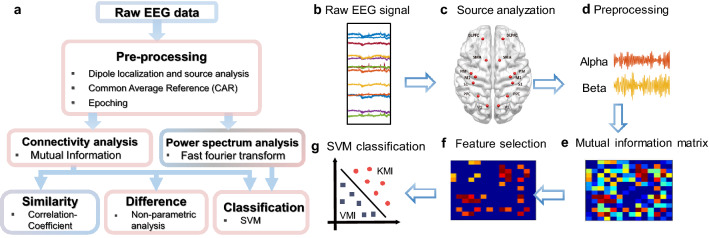
A schematic diagram of a brain network classification using mutual information. (**a**) A schematic procedure illustrating the overall workflows. (**b**) Raw EEG signal. (**c**) ROI location and source analysis. (**d**) Signal preprocessing (e.g., band-pass filtering and epoching). (**e**) Calculate mutual information at each frequency band. (**f**) Feature selection with a threshold of 0.2. (**g**) Classification of the KMI and VMI using SVM.

We selected 14 areas related to cognitive mechanisms of arm movement processes, which were expected to be involved in the ME, KMI, VMI, and VO networks^31–34^ in the bilateral hemispheres as follows: the dorsolateral prefrontal cortex (DLPFC), premotor cortex (PM), supplementary motor area (SMA), primary motor cortex (M1), primary somatosensory cortex (S1), posterior parietal cortex (PPC), and primary visual cortex (V1). The locations are illustrated in Fig. 2c. We performed source analysis according to the determined region of interest (ROI) using the built-in function of the discrete model probing in BESA research 6.0 (GmbH, GrmbH, Gru, Bavaria, Germany). The EEG signals, which are illustrated in Fig. 2b, were band-pass filtered at a range of 0.1–100 Hz with a 60 Hz notch filter (Fig. 2d). Table 1 lists the coordinates used^35^. Each source was identically analyzed for all subjects by using equal masks.

**Table 1:** The regions of interest (ROIs).

#	ROIs	Talairach coordinate		
		x	y	z
1	Left dorsolateral prefrontal cortex (DLPFC)	− 17.82	46.13	29.01
2	Left supplementary motor area (SMA)	− 9.9	8.62	55.76
3	Left premotor cortex (PM)	− 37.3	− 14.4	60.3
4	Left primary motor cortex (M1)	− 34.65	− 20.4	58.13
5	Left primary sensory cortex (S1)	− 35.64	− 32.07	57.8
6	Left posterior parietal cortex (PPC)	− 25.74	− 54.35	58.91
7	Left primary visual cortex (V1)	− 9.9	− 71.32	10.94
8	Right dorsolateral prefrontal cortex (DLPFC)	17.82	46.13	29.01
9	Right supplementary motor area (SMA)	9.9	8.62	55.76
10	Right premotor cortex (PM)	37.3	− 14.4	60.3
11	Right primary motor cortex (M1)	34.65	− 20.4	58.13
12	Right primary sensory cortex (S1)	35.64	− 32.07	57.8
13	Right posterior parietal cortex (PPC)	25.74	− 54.35	58.91
14	Right primary visual cortex (V1)	9.9	− 71.32	10.94

Before the source analysis, the common average reference (CAR) was used to remove the global background activity^36^. We separated the ROI signal into theta (3–7 Hz), alpha (8–12 Hz), beta (13–30 Hz), and gamma (32–100 Hz) frequency bands.

### Power spectral density analysis

The source-localized and filtered data were epoched with a time window from 4 s before the onset to 13.2 s after the onset. To calculate the power for each condition, we used the Morlet wavelet transform with a frequency range of 0.1–100 Hz. The power spectra from the task time (0–8.2 s) were normalized by the power of each frequency from the baseline periods (− 1.5 to − 1 s). Then, the data were averaged for all ROIs making one power spectra for each trial. To compare the difference between conditions, we made one grand averaged power spectra plot for each condition by averaging the normalized power spectra of all the trials and subjects at each condition.

### Connectivity analysis

To compare the patterns of two MIs, we analyzed brain connectivity using a mutual information algorithm (Fig. 2e). Mutual information measures both linear and nonlinear interdependencies between EEG signals. We used cross mutual information, which can be applied to two different signals^37^. In this study, we utilized mutual information between the signals at the paired cortical ROIs. The connectivity values from the task time (0–8.2 s) were normalized by the connectivity values of each session from the baseline periods (− 1.5 to − 1.4 s). We had a total of 91 edges (14 ROIs), and each condition (i.e., ME, KMI, VMI, and VO) had 48 trials per individual. Connectivity patterns were grand averaged across subjects for the four conditions with the alpha and beta frequency bands separately.

### Network analysis with graph theory

Graph theory was used to reconstruct the brain network. We used undirected weighted values from the connectivity with mutual information as the network edges. We tried to identify the critical nodes that have strong relationships with other nodes in the network. We estimated the degree centrality (DC) in the weighted graph with the GRETNA toolbox^38^. The calculated DC was represented for each frequency band.

### Degree centrality

The DC of a node is can be easily computed by summing the weight of edges incident on the node. To analyze DC, we used gretna_node_degree_weight (GRETNA). If A is the N × N weighted matrix for a graph, the DC of the *i*-th node is quantitatively defined as follows:

$${\text{DC}}(i) = \mathop \sum \limits_{j = 1}^{i - 1} A[i,\;j] + \mathop \sum \limits_{j = i + 1}^{n} A[i,\;j]$$

where N is the number of nodes.

### Feature extraction and classification

Support vector machine (SVM) analysis was used to classify the KMI and VMI in a single trial. We used network measures and normalized power as features to classify the KMI and VMI to compare the classification accuracy of each measurement. The features were used by the edges, which consisted of the highest 20%^39^ (Fig. 2f), and normalized power, respectively. To classify with the SVM algorithm, we used the MATLAB function of fitcsvm (Fig. 2g). The classification performance was evaluated by a fivefold cross-validation with 100 iterations. The overall procedure is illustrated in Fig. 2a.

### Statistical analysis

The significant difference between the degree centralities at the nodes for KMI and VMI was calculated for each frequency band using a non-parametric permutation test. The significance was evaluated by the permutation distribution with 10,000 iterations^40^. Pearson's correlation coefficient (R) was calculated to measure the similarity between the connectivity matrixes of the different conditions. All analyses were conducted with MATLAB (R2018a, Math-Works, Natick, MA, USA) and the EEGLAB toolbox^41^.

## Results

### Connectivity analysis using mutual information

We analyzed the connectivity patterns for each condition in the four frequency bands, but the theta and gamma frequency band signals did not produce statistical differences between the ROI signals. Therefore, the results focused on the alpha and beta frequency signals, and the remaining two frequency results are not given. There was a total of 14 ROIs, and the time series of one trial was 8.2 s for each subject. The window size was 100 ms, which was the same as in our previous study^42^, with no overlap. Eighty-two windows were created per trial. Each ROI signal was analyzed with the mutual information algorithm, which yielded one 14-by-14 matrix for each time point of the windows. These 82 connectivity matrices generated at each time point were averaged to create one 14-by-14 matrix per trial. The connectivity matrix of all the trials was finally averaged for each condition, resulting in one 14-by-14 connectivity matrix.

### Connectivity classification using SVM

**Figure 3:**
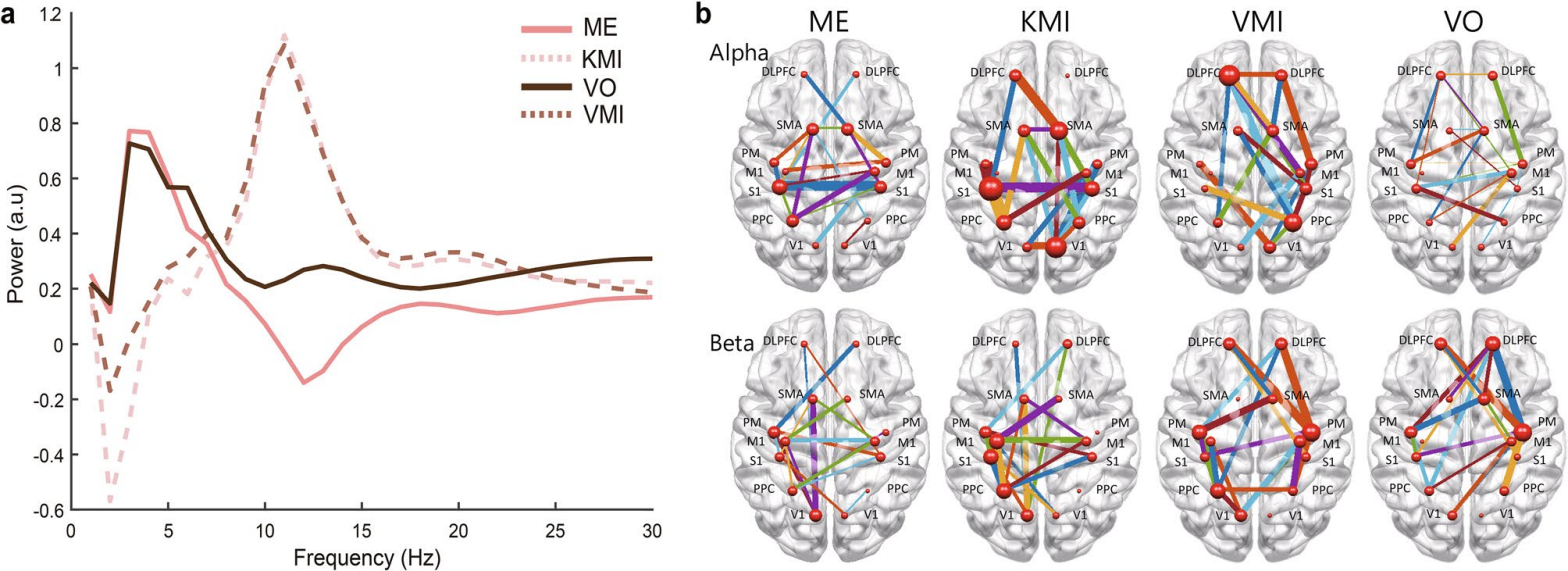
An example of the features for classifying the KMI and VMI. (**a**) The Grand averaged power spectra of the four conditions. Each line indicates the normalized power for each condition grand averaged across all subjects and ROIs. For the further classification of KMI and VMI, we used the single trial-normalized power for the features (*a.u* arbitrary unit). (**b**) Grand averaged Highest 20% connections (as SVM features) at each frequency bands for each condition. The edges represent the functional connectivity calculated by the mutual information, and the thickness of the edge indicates the strength of the connectivity. The nodes represent the locations of the ROIs, and the different sizes of the node express the DC, that is, how many links are connected to that node.

**Figure 4:**
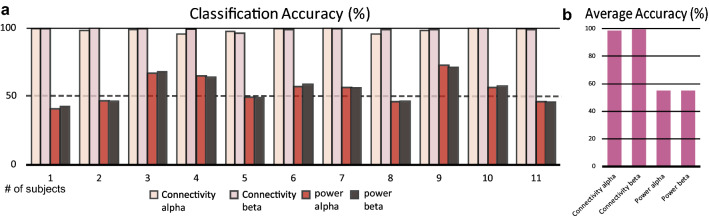
The average classification accuracy. (**a**) The individual classification accuracy of eleven subjects by the measure of connectivity and power. Dashed black line indicates the chance level (50%). (**b**) The averaged classification accuracy in the features of connectivity and power at the alpha and beta frequency bands. The average accuracy of each feature: connectivity-alpha (98.52 ± 1.57), connectivity-beta (99.28 ± 1.04), power-alpha (54.95 ± 10.11) and power-beta (55.10 ± 9.72).

**Figure 5:**
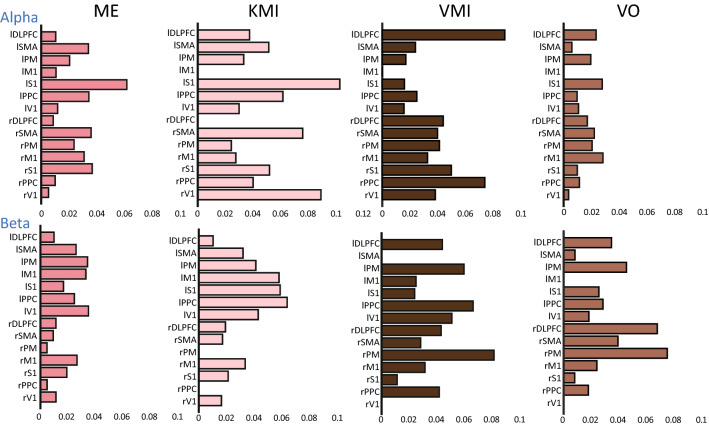
Node degree centrality (NDC) of each condition at the alpha and beta frequency. The NDC was calculated from the grand averaged edges with a threshold of 0.2. The NDC of each condition corresponds to the size of the nodes in Fig. 3b. *l* left, *r* right.

We used the SVM algorithm to classify the KMI and VMI based on the two different feature groups. One is the power spectrum, which is normalized by the baseline power, and the other is the connectivity. We compared the classification performances. Figure 3a is an example of the features for classifying the KMI and VMI. Each line represents the normalized power spectra grand averaged across all subjects for each condition. We used the normalized power of the alpha and beta frequency as the features for the SVM. The normalized power of the KMI and VMI conditions was similar between the alpha and beta frequency bands (R = 0.99), which could explain the low classification performance when using the normalized power shown in Fig. 4. Figure 3b depicts the highest 20% of the grand averaged connectivity at the alpha and beta frequency bands under each condition. The edge was calculated by mutual information algorithms, and all trials were averaged for all subjects. The size of a node represents the node degree centrality. The edges shown in Fig. 3b were selected for the features to classify the KMI and VMI conditions. Note that the networks of the KMI and ME had more connections to the ROI of the sensorimotor cortex and showed similar network patterns. On the other hand, the network patterns of the VMI and VO were similar, showing many networks at the DLPFC and PM (Table 3; Fig. 5). The classification was performed based on individual trials using the extracted features. The classification accuracies between the KMI and VMI are shown in Fig. 4a. In Fig. 4a, the accuracy was higher in the connectivity feature than in the power feature. In addition, the individual's accuracy was higher than the chance level (50%). The averaged accuracy was 98.5% in the alpha and 99.29% in the beta band with the connectivity features (Fig. 4b). On the other hand, the classification accuracies with the features of the power spectrum were significantly lower (p < 0.01, Wilcoxon paired rank test) than the accuracy with the connectivity features (alpha: 54.95%; beta: 55.1%).

To strengthen the results of the present study, we classified the ME and KMI and VO and VMI by using connectivity measures as a classifying feature (Table 2). The classification accuracy for each participant was slightly higher than the chance level (50%). The accuracy of mean and standard deviation of the ME and KMI was (62.9 ± 19.7) and (62 ± 15) for the alpha and beta frequency bands, respectively. The mean accuracy of the VO and VMI was (57 ± 14) and (54 ± 16.8) for the alpha and beta frequency bands, respectively. This is an important result of supporting our hypothesis because we hypothesized that motor imageries share a similar sensory modality to the equivalent actual movements.

**Table 2:** The classification accuracy between the ME and KMI or VO and VMI by using connectivity measures as a classifying feature.

Subject	ME-KMI		VO-VMI	
	Alpha	Beta	Alpha	Beta
1	84	76	46	47
2	86	46	49	41
3	58	63	55	42
4	45	49	62	43
5	49	60	58	55
6	42	41	47	44
7	42	61	55	68
8	64	58	50	40
9	79	68	63	50
10	98	99	98	98
11	44	63	47	67
Mean ± SD	62.9 ± 19.7	62 ± 15	57 ± 14	54 ± 16.8

### Correlation of connectivity patterns between KMI and VMI

The Pearson’s correlation of connectivity patterns between conditions was analyzed to identify global similarity. Table 3 shows the results of the correlation coefficient between the conditions. Correlation coefficients were calculated from the grand average of the connectivity matrix. The correlation coefficients between the KMI and ME and between the VMI and VO were significantly higher (p < 0.01) in the alpha and beta frequency bands, respectively. The similarity between the KMI and ME verifies our hypothesis that the brain network of the KMI is close to that of the ME. This is also the reason why the ME and KMI or VO and VMI cannot be separated by using connectivity measures as a classifying feature, respectively, as shown in Table 2. On the other hand, the other pairs were not significant. In particular, the insignificant correlation between the KMI and VMI was quite reasonable because the two connectivity patterns were able to be discriminated with high accuracy.

**Table 3:**
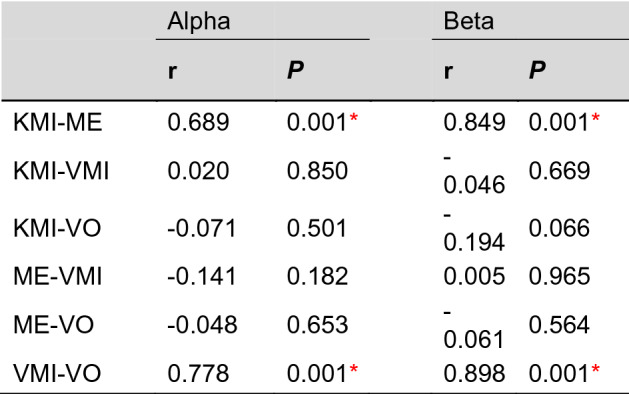
The network correlation coefficients between each condition at the alpha and beta frequency bands, respectively. *p < 0.01.

r denotes the Pearson's correlation coefficient.

### Degree centrality of the KMI and VMI

**Figure 6:**
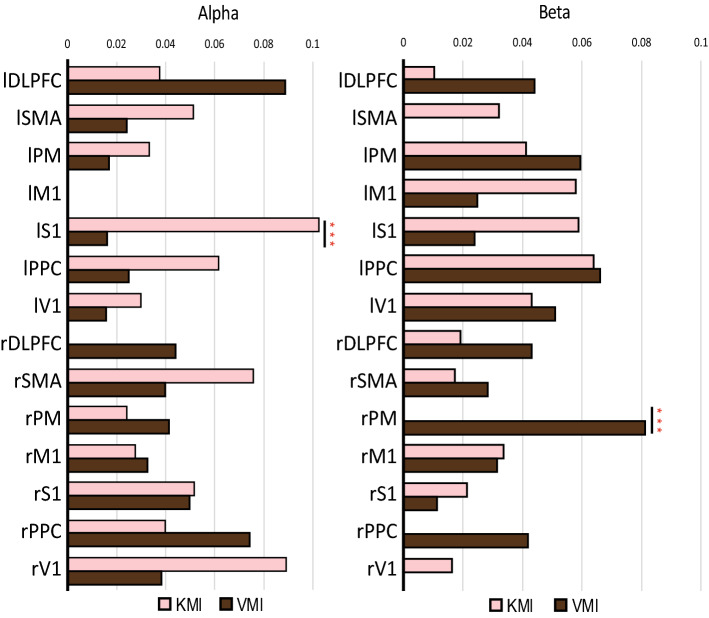
The significant difference in the NDC of the KMI and VMI at each frequency band. At the alpha frequency, the KMI was significantly higher than the VMI at the left S1. On the other hand, the right PM was higher than the KMI. p-value was calculated with the non-parametric permutation test. ***p < 0.05.

We further measured the DC with the connectivity values in the brain network shown in Fig. 5. The top 20% of the connectivity values were selected for the network measure. In graph theory, the DC on a node is measured by adding the connectivity values from the node to the other nodes in the network. Here, we used DC to find out the local difference between the KMI and VMI in the alpha and beta frequency bands. Figure 6 depicts the overall DC in the alpha and beta frequency bands, which shows significant differences (*p* < 0.05) between the KMI and VMI. The result shows that the alpha frequency band showed significant differences (*p* < 0.05) in the left S1, and the beta frequency band showed significant differences (*p* < 0.05) in the right PM.

## Discussion

This study investigated the characteristics of two major MI types, KMI and VMI, by using connectivity patterns and a network measure. The results show that the KMI and VMI could be classified in the SVM method on single trials with high accuracy. To the best of our knowledge, the classification with the connectivity patterns is the first applied in discriminating the type of MI. The reason for the excellent classification performance is that the cognitive brain networks of the KMI and VMI would be different.

The results of this study showed similar network patterns between the ME and KMI and between the VMI and VO, respectively. These large network similarities show the underlying neural networks and neural mechanisms of different cognitive conditions because mental imagery uses almost identical neural substrates as perception in the same sensory modality^43^. In this case, we used an experimental design from the perspective that motor imageries maintain the same temporal characteristics as the equivalent actual movement^44^ and facilitate the receptive imagery task^14,29^. Therefore, these facilitations of the cognitive system at the cortical level would be the reason for the global network similarities between the KMI and ME and between the VMI and VO, respectively. In particular, the similarity between the KMI and ME implies the effectiveness of utilizing a decoder trained by an actual movement.

The local DC at a ROI level reflects the relationship of the ROI with other ROIs. Thus, key ROIs in each condition would have high values in the local DC. In the comparison of DC between the KMI and VMI, the left (contralateral side) S1 was significantly higher in the KMI than in VMI at the alpha band. The S1 is known as the primary region for somatosensory perception including proprioception. According to some reports, subjects improved their ability for MI by giving sensory stimulation during the task. This MI improvement suggests that through the processing of information by the neural circuitry associated with MI, sensory signals trigger a motoric memory of a given movement and support body limb representation^45,46^. Thus, S1 in KMI should be more activated and centralized for kinesthetic sensation than in VMI. In addition, in the beta frequency band, the DC of the right (ipsilateral) PM in VMI was significantly higher than in KMI. It is known that the premotor cortex could be activated in movement imagery, which involves planning and preparation for actual movements^47^. Another fMRI study showed that just observing a movement could facilitate motor learning by mirror neurons in the premotor cortex^48^. Thus, the PM might be associated with an action observation and would have an important impact on the VMI similar to the VO.

A previous study classified four MI conditions by the power amplitude^14^. That study found that event-related spectral perturbation (ERSP) patterns between the ME and KMI and between the VMI and VO were also similar. In particular, KMI is similar to ME because KMI increases the corticospinal excitability and is related to sensorimotor systems^49^. Additionally, the ERD pattern of the KMI was similar to that of actual executions^50^, while the KMI and VMI were mediated through separate neural systems^16^. However, a meta-analysis found no significant contrast from the triggered anatomical regions of the KMI and VMI in sports experts^33^. Moreover, some studies reported that the activity of VO was similar to ME because the activated patterns and sequences of the brain were similar to the real executions^30,51^. Due to this discrepancy, the classification of cognitive conditions by brain activation like ERSP has limitations. In our study, classification with band powers resulted in a worse performance than that with the connectivity features.

Instead of the activation feature, the neural pathway would be an alternative feature. Our previous studies demonstrated that the connectivity increased during movement execution independent of the strength of the brain activity^20,42^. The present results also show that connectivity measure is more powerful in classifying the type of MI than by the power measure. The complex cognitive process involves multiple brain regions. Thus, we used the brain source localization to extract representative source activity for the determined ROIs. Because neighbor channels in the EEG show a similar activity, features of the connectivity based on the activity of the channel level might not be effective. Relatively sparse source ROIs could result in a high classification accuracy. The neuronal activity does not occur alone. It has a relationship with other neurons. The connectivity evaluates the relation between the ROIs. This relationship is reflected in the connectivity-based feature. Therefore, these edges provide valuable features to classify KMI and VMI.

It is still difficult to train KMI and VMI performance. In particular, the people who cannot move their hands because of paralysis would have difficulty to incorporate an accurate MI. However, the training of a MI similar to an actual execution is essential. A MI is a covert action, which is in the category of exercise, and giving feedback is essential to improving the imagery ability. Our study shows that the strategy of MI, such as KMI and VMI, can be classified with very high performance. The network pattern of KMI is similar to that of real execution. The BMI should give sensory feedback to improve the performance of future BMIs. Combining kinesthetic motor imagery with somatosensory feedback would make a more vivid motor imagery, which is similar to the networks of movement execution.

Although MI training is important, there is no promising training protocol yet. Considering our results, it is possible to provide KMI feedback similar to actual movement through brain connectivity analysis. In addition, each individual can be given feedback to see if the imagination is similar to the natural signal of the actual movement. Future research will have to be done on how the network patterns of ME and KMI are so similar. In addition, future motor imagery training should not only provide movement information but also provide sensory feedback and proprioception information when moving so that it feels like a real move.

There are some limitations to the study. One of the current limitations is the small number of subjects in our study. However, the accuracy of classifying the KMI and VMI in a single trial showed that the possibility of giving accurate feedback is high. And the trial number of each individual was enough to permit statistical analysis showing differences between the KMI and VMI. Another limitation is that it is difficult to verify whether the subjects were performing KMI and VMI in the correct manner. In order to address this problem, KVIQ-10 was used to access the individual imagery ability to perform MI prior to the experiment, and it was confirmed that no subjects excluded from the criteria. Additionally, we used a block design to facilitate each imagery. This facilitation involves reproducing previously experienced movements from memory^52^, which helps guide subjects to use each imagery correctly.

## Conclusion

Classification of two motor imageries was significantly better with the features of connectivity than with normalized powers. Consequently, the high classification could be used as feedback for MI training. Because the KMI network characteristics were similar to ME, KMI would be more suitable for BMI. S1 in the alpha frequency band and right PM in the beta frequency band are the key nodes in KMI and VMI, respectively. These results open up new perspectives for the underlying neural networks of the cognitive conditions and toward designing MI training methods.
